# Association of Circulating Tumor Cells, Megakaryocytes and a High Immune-Inflammatory Environment in Metastatic Breast Cancer

**DOI:** 10.3390/cancers15133397

**Published:** 2023-06-28

**Authors:** Cvetka Grašič Kuhar, Jernej Silvester, Marina Mencinger, Tanja Ovčariček, Maja Čemažar, Simona Miceska, Živa Modic, Anamarija Kuhar, Tanja Jesenko, Veronika Kloboves Prevodnik

**Affiliations:** 1 Department Medical Oncology, Institute of Oncology, 1000 Ljubljana, Slovenia; 2 Faculty of Medicine Ljubljana, University of Ljubljana, 1000 Ljubljana, Slovenia; 3 Department of Experimental Oncology, Institute of Oncology, 1000 Ljubljana, Slovenia; 4 Faculty of Health Sciences, University of Primorska, 6000 Izola, Slovenia; 5 Department of Cytopathology, Institute of Oncology, 1000 Ljubljana, Slovenia; 6 Faculty of Medicine, University of Maribor, 2000 Ljubljana, Slovenia

**Keywords:** breast cancer, circulating tumor cell, cluster, megakaryocyte, pan-inflammatory value, overall survival

## Abstract

**Simple Summary:**

In the present work on metastatic breast cancer patients, we studied liquid biopsy samples to analyze different biomarkers, such as circulating tumor cells, leukocytes, and platelets. We observed a correlation between a higher number of circulating tumor cells and a greater probability of detecting clusters and megakaryocytes among the patients. This observation was independent of the breast cancer subtype. We also confirmed the presence of megakaryocytes in the peripheral blood of cancer patients, which is an unusual finding. In addition, a higher pan-inflammatory value, calculated by dividing the product of neutrophils, monocytes, and platelets by the number of lymphocytes, was associated with lower overall survival, providing insight into the depth of the metastatic process.

**Abstract:**

Liquid biopsy is becoming an important source of new biomarkers during the treatment of metastatic cancer patients. Using size-based microfluid technology, we isolated circulating tumor cells (CTCs) from metastatic breast cancer patients to evaluate their presence and cluster formation, as well as the presence of megakaryocytes and immune-inflammatory blood cells, and to correlate their presence with clinicopathological data and overall survival (OS). In total, 59 patients (median age 60.4 years) were included in the study: 62.7% luminal A/B-like, 20.3% HER2-positive, and 17% triple-negative. Our results showed that at least one CTC was present in 79.7% and ≥5 CTCs in 35.2% of the patients. CTC clusters were present in patients with ≥5 CTCs only (in 19.2% of them), and megakaryocytes were present in 52% of all patients. The presence of CTC clusters and megakaryocytes was positively associated with the CTC count. Patients with low pan-inflammatory value (PIV), low systemic immune-inflammatory index (SII), and low relative change from baseline (ΔPIV%, ΔSII%) were associated with significantly higher OS than their counterparts. ΔPIV%, the presence of infection in the last month, and a long duration of metastatic disease were identified as independent prognostic factors for OS. The interplay of CTCs, CTC clusters, megakaryocytes, and PIV needs to be further explored.

## 1. Introduction

Metastatic breast cancer remains an incurable disease and continues to be the leading cause of cancer-related deaths in women in Western countries due to its high incidence [1]. Metastases can occur at any stage of the disease, either early or late, and some patients are already diagnosed with metastatic disease at presentation. The location of metastases (bone vs. visceral), molecular subtype (based on hormonal receptors, HER-2 status, grade, and Ki-67 index) [2], comorbidities, and performance status are well-established prognostic factors for overall survival (OS). However, there is a need for novel noninvasive predictive and prognostic biomarkers that could more precisely predict the response to treatment and OS. In clinical practice, an elevation of certain peripheral blood parameters, such as CA 15-3, lactate dehydrogenase, or leukocytosis, can serve as predictive indicators for the appearance of resistant disease clones. Currently, the most commonly utilized method for assessing treatment response is a radiological evaluation based on the Response Evaluation Criteria in Solid Tumors (RECIST). However, this approach has limitations, particularly in regard to evaluating non-target lesions [3], and it may not promptly identify the emergence of resistant clones, leading to the continuation of ineffective treatment and delaying appropriate treatment adjustments. Moreover, patients with advanced metastatic disease are often in poor general condition to undergo repeated invasive diagnostic procedures for tumor analysis. Therefore, biomarkers for monitoring treatment response in a minimally invasive way are urgently needed.

On the other hand, liquid biopsy is a source of novel prognostic markers for different cancers, and it can be easily collected in a patient-friendly manner. Circulating tumor cells (CTCs) are tumor cells that diverge from the primary tumor or distant metastases and enter the circulation. They exist in the circulation only transiently, as they either die or become trapped in the capillaries of a distant organ [4]. Pooled analysis of retrospective data in 2436 metastatic breast cancer patients demonstrated the independent prognostic role of CTCs for OS. Patients with ≥5 CTCs per 7.5 mL of peripheral blood were characterized by a shorter median OS than patients with <5 CTCs [5]. However, due to the dynamic nature of CTC shedding into the bloodstream, the absence of CTCs does not necessarily indicate a less aggressive disease [6]. Interestingly, there is one study reporting more CTCs in blood samples of patients during sleep than during the active phase, and those CTCs were associated with augmented metastatic potential [7].

Inflammation is one of the hallmarks of cancer development and progression [8]. Local and systemic inflammation is associated with decreased survival in cancer patients [9]. The immune cell count in the peripheral blood reflects systemic inflammation. It has been reported that a high neutrophil count inhibits the immune system by suppressing the cytolytic activity of immune cells, including lymphocytes, and natural killer cells, thereby promoting cancer progression [10]. Furthermore, various immune-inflammation scores can be calculated from the complete blood count as well. For example, a high neutrophil-to-lymphocyte ratio has been linked to a poor prognosis [11], and a high systemic immune-inflammation index (SII) has been associated with worse OS in numerous solid tumors [12]. In early breast cancer, a high SII determined prior to neoadjuvant chemotherapy has been associated with decreased OS [13]. Recently, a new prognostic index, pan-immune-inflammation value (PIV) has been shown to have prognostic value for OS in operable breast cancer [14].

Moreover, some blood cells, such as neutrophils and platelets, could serve as partners in the formation of heterotypic clusters of CTCs, which have 20–100× greater metastatic efficiency than individual CTCs [15,16,17]. The role of megakaryocytes in cancer progression has not yet been established. Normally, megakaryocytes are placed in bone marrow, where they are involved in platelet production, and they are rarely present in the peripheral blood of nonhematological diseases [18]. Furthermore, there are some reports showing that megakaryocytes may also have an immune function [19]. However, despite many encouraging results, the determination of CTCs and the calculation of immune-inflammatory markers have not yet been introduced into routine clinical practice. Our earlier investigations [20,21] have enabled us to identify the most suitable method for harvesting CTCs using size-based microfluid technology. In the present study, we conducted further cytopathological research on isolated cells from metastatic cancer blood samples: CTCs, clusters, and megakaryocytes. Our objective was to investigate the potential associations between CTCs, megakaryocytes, and immune/inflammatory cells with the clinical characteristics of metastatic breast cancer patients and to assess their prognostic significance for OS.

We hypothesized that parameters, such as a higher CTC count, the presence of megakaryocytes, and high SII and PIV values, would be negatively associated with patient OS.

## 2. Materials and Methods

We conducted a noninterventional prospective study involving 59 patients with metastatic breast cancer. Patients were enrolled regardless of the duration of the disease and the line, and the cycle of therapy. We correlated the number of CTCs, the number of megakaryocytes, and blood-based immune-inflammatory markers with the clinical characteristics of the patients. Additionally, we performed an analysis of potential biomarkers for OS. Relevant clinical and pathological data were obtained from patients’ medical records. All patients provided written consent. The study protocol was approved by the Institutional Ethics Committee (ERIDNVPO 0021/2020) and Slovenian National Ethics Committee (0120-541/2021/3). The study was conducted in accordance with the Helsinki Declaration and Good Clinical Practice.

CTCs were isolated from 10 mL of peripheral blood collected into EDTA tubes using the Parsortix^®^ method (Angle, Guildford, UK) according to the manufacturer’s recommendations. The Parsortix^®^ separation cassette contains a stepped structure, gradually narrowing in diameter until reaching a final gap of 6.5 μm. Therefore, all of the cells that are larger than 6.5 μm are retained and isolated. Retained cells were harvested into a 5 mL plain red-top vacutainer tube (Becton Dickinson, Franklin Lakes, NJ, USA) without a preharvest flush and resuspended in an in-house cell medium: 20% bovine serum albumin (SERVA, Heidelberg, Germany), 5% EDTA (Sigma Aldrich, St. Louis, MI, USA) in PBS [21].

The suspension of isolated CTCs and megakaryocytes was centrifuged on slides by cytocentrifuge (Thermo Scientific Shandon Cytospin^®^ 4 Cytocentrifuge, Waltham, MA, USA) at 700 rpm for 4 min at room temperature, and four cytospins were prepared in total. The first cytospin was air-dried at room temperature for at least 30 min and later stained by Giemsa (Lopez Cordosa Giemsa, Sigma Aldrich, Merck (Darmstadt, Germany) on a Leica automated slide stainer XL (Leica Microsystems, Buffalo Grove, IL, USA). Giemsa cytospin was used for microscopic examination and cell counting, as previously described [21]. The second cytospin was fixed in Delaunay (2500 mL acetone, 2500 mL absolute ethanol and 2.5 mL 1 M trichloroacetic acid) for at least 30 min and stained by Papanicolaou (PAP) on a Leica automated slide multistainer ST5020 (Leica Microsystems, Buffalo Grove, IL, USA) and was used for immunocytochemical (ICC) staining with anti-cytokeratin antibody (CK AE1/AE3, M3515, 1:100, Agilent, Santa Clara, CA, USA). The other two cytospins were fixed in methanol and were also used for ICC staining with anti-megakaryocyte antibody (CD61, clone 2f2; 1:50, Cell Marque, Rocklin, CA, USA) and anti-macrophage antibody (CD68, clone KP1, 1:200, Agilent). ICC staining was performed on a BenchMark ULTRA automated immunostainer (Roche Diagnostics, Rotkreuz, Switzerland), and the staining protocols were a part of the standardized ICC protocols at the Institute of Oncology Ljubljana [22].

The Interaction between inflammatory pro-cancer populations (i.e., neutrophils, platelets and monocytes) and anticancer immune populations (i.e., lymphocytes) was assessed by SII and PIV indexes. SII and PIV indexes were calculated as follows: SII = (P × N)/L, and PIV = (P × N × M)/L, where P, N, M, and L were absolute counts of platelets, neutrophils, monocytes, and lymphocytes in a milliliter of peripheral blood. The optimal cutoff value between ‘low’ and ‘high’ SII and PIV was set from the receiver operator curve (ROC curve) for OS. We used these cutoffs after checking their credibility in previous studies [13,14,23,24].

Relative changes in SII and PIV were defined as ΔSII% and ΔPIV% and calculated as ΔSII% = ((SII_at CTC collection_ − SII_at first metastatic presentation_)/SII_at first metastatic presentation_) and ΔPIV% = ((PIV_at CTC collection_ − PIV_at first metastatic presentation_/PIV_at first metastatic presentation_). These calculations were adopted from the methodology defined by Shang et al. [25]. The optimal cutoff value between ‘low’ and ‘high’ values was calculated from the ROC curve for OS.

Blood samples used to determine the immune cells were obtained on the same day and in the same setting as a sample for CTCs. All blood samples were analyzed on Sysmex XN-Series analyzer (Sysmex Europe, Norderstedt, Germany).

Patient characteristics are described as frequencies and percentages for qualitative data and medians (±standard deviations) and ranges or interquartile ranges for quantitative data. Breast cancer molecular subtypes were based on the clinicopathological surrogate definition [2]. The association between demographic and clinicopathological data and CTC count, megakaryocytes, platelet volume, SII, PIV, ΔSII%, and ΔPIV% was performed by means of Pearson’s chi-square or Fisher’s exact test. We compared the possible association of clinicopathological parameters with the number of CTCs among groups of patients having 0 CTCs vs. ≥1 CTCs and between 0 CTCs vs. 1–4 CTCs vs. ≥5 CTCs. As we performed CTC harvesting from a total of 10 mL of blood, the number of CTCs per 10 mL was converted to the number of CTCs per 7.5 mL of blood. This conversion was based on the standard definition established in the literature [5]. Megakaryocyte analysis was performed based on two groups: 0 vs. ≥1 megakaryocyte. OS was calculated as the time from CTC collection to death or last follow-up. Kaplan–Meier curves with the log-rank test were used to evaluate OS. A Cox proportional hazards regression model was used for univariate and multivariate analyses. A value of *p* ≤ 0.05 was considered statistically significant. Statistical analysis was performed using SPSS v.24.

## 3. Results

### 3.1. Patient Population

**Figure 1:**
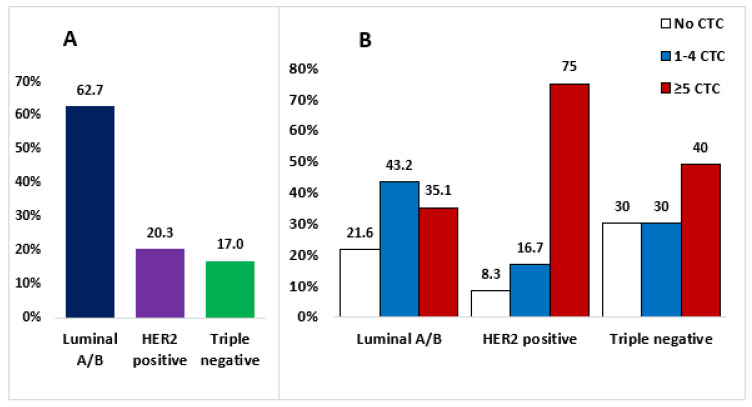
(**A**). Distribution of molecular subtypes in our patient cohort. (**B**). Percentage of patients with 0, 1–4 and ≥5 CTCs in 7.5 mL of peripheral blood based on the molecular subtype of breast cancer.

Whole blood samples of 59 patients with metastatic breast cancer were drawn to isolate CTCs. The median age of patients at diagnosis was 50.3 years and 60.4 years at CTC collection. The majority of patients (81.3%) had invasive ductal carcinoma, 61% had grade 3, and 62.7% belonged to luminal A-like and luminal B-like. Additionally, 20.3% of the patients were HER2-positive (HER2+), and 17% were classified as having the triple-negative subtype (Figure 1A). Seventy-eight percent of patients underwent surgical treatment, 57.6% underwent adjuvant chemotherapy, and 64.2% received adjuvant endocrine therapy. Twenty-two percent (12 patients) were diagnosed with primary metastatic cancer (58.3% luminal A/B-like, 16.7% HER2+, 25% triple-negative) and did not undergo surgery. Detailed characteristics of patients with breast cancer at diagnosis are presented in Table S1.

#### 3.1.1. CTCs

**Figure 2:**
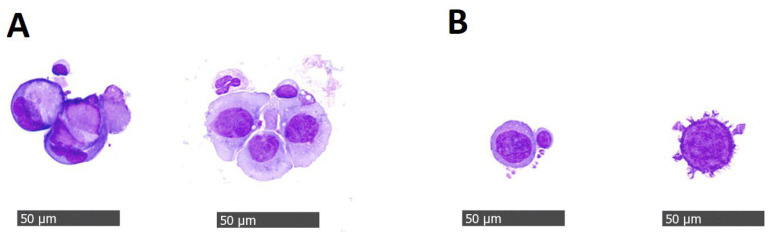
Representative image of (**A**) CTC clusters and (**B**) single CTCs.

In 47 (79.7%) patients, at least one CTC was detected. In the luminal A/B-like subtype, 43.2% of patients had 1–4 CTCs/7.5 mL of blood, 35.1% had ≥5 CTCs/7.5 mL of blood, and in 21.6% of patients, CTCs were not found in the blood samples. In the HER2+ subtype, 16.7% of the patients had 1–4 CTCs, 75% of them had ≥5 CTCs, and 8.3% had 0 CTCs. In the triple-negative subtype, 30%, 40%, and 30% of patients had 1–4 CTCs, ≥5 CTCs, and 0 CTCs, respectively (Figure 1B). There was no significant difference in CTC count in different molecular subtypes. However, in the HER2+ subtype more patients (three quarters) had ≥5 CTCs than in the luminal A/B and triple-negative subtypes. Clusters of CTCs (Figure 2) were found in 5 (8.5%) patients (two with luminal A/B and three with HER2+ subtype). Clinical data and isolated cell characteristics, compared in the groups of patients with 0 CTCs, 1–4 CTCs, and ≥5 CTCs are presented in Table 1. To summarize, CTC clusters were significantly associated with the CTC group and were present only in the group of patients characterized by ≥5 CTCs (19.2% of them also had clusters present). Furthermore, we observed a moderate positive correlation between the number of CTCs and clusters (Pearson’s r = 0.416 (95% CI 0.179–0.608); *p* = 0.001). Similarly, megakaryocytes seemed to be more often present in patients with more CTCs. Patients in different CTC groups did not exhibit any significant differences based on the parameters, such as type of treatment, line of therapy, duration of metastatic disease, level of immune-inflammatory markers SII, PIV, ΔSII%, and ΔPII%.

#### 3.1.2. Megakaryocytes

**Figure 3:**
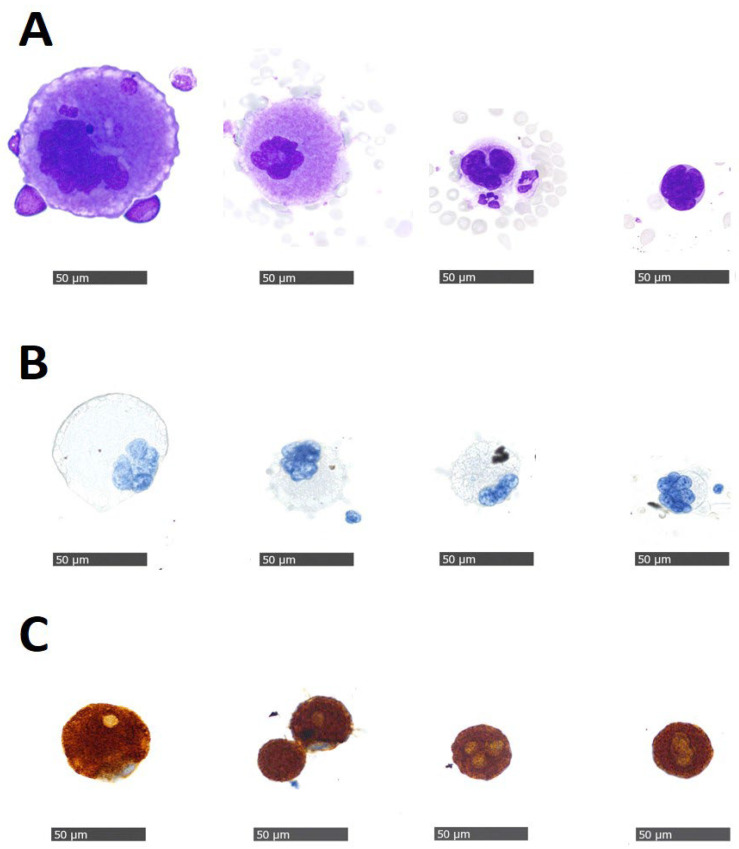
Representative images of megakaryocytes found in the peripheral blood of metastatic breast cancer patients. (**A**) Giemsa staining, (**B**) immunocytochemical staining for macrophage marker CD68 (no positive staining was observed), (**C**) immunocytochemical staining for megakaryocyte marker CD61 (brown color (DAB) indicates positive staining) (×400 magnification).

Evaluation of CTCs on Giemsa slides indicated the presence of cells with morphological features of megakaryocytes in 31 patients (52.5%), which was not expected. Their phenotype was confirmed by ICC staining with anti-CD61 antibody in 5/5 cases and by negative staining with anti-CD68 and anti-cytokeratin antibodies. (Figure 3). Megakaryocytes were detected in 27 samples with CTCs and in 4 patients without any CTCs present in the blood samples. Moreover, we analyzed the possible association of the presence of megakaryocytes with molecular subtypes of breast cancer, treatment, number of CTCs, CTC clusters, and immune-inflammatory markers (Table 2). Patients with megakaryocytes showed no significant differences associated with the subtype, length of metastatic disease, line of treatment, or type of therapy (chemotherapy or endocrine therapy) compared to patients for which we did not detect any megakaryocytes. However, we observed a moderate positive correlation between the number of megakaryocytes and the number of CTCs (Pearson’s r = 0.449 (95% CI 0.211–0.637); *p* < 0.001; Figure S1). All patients with CTC clusters were also characterized by the presence of megakaryocytes in the blood samples, although our results indicated a weak correlation (Spearman’s rho = 0.289 (95% CI 0.028–0.513); *p* = 0.026). Patients with high PIV values at CTC collection, along with high ΔSII% values, were also found to be more likely to have megakaryocytes.

#### 3.1.3. Blood-Based Immune-Inflammatory Markers SII, PIV, ΔSII%, and ΔPIV%

The optimal cutoff value between the ‘low’ and ‘high’ PIV values was set at 368 and between ‘low’ and ‘high’ SII values at 840 × 10^9^ (Figure S2A,B). The association of the PIV category (low and high) with clinicopathological features, CTCs, and megakaryocyte count is presented in Table 3. The distribution on low and high PIV did not differ among the molecular subtypes. The analysis revealed an association of low PIV with skeletal metastases and treatment with endocrine therapy. On the other hand, high PIV was present in patients who underwent chemotherapy and in patients with megakaryocytes in their blood. The SII marker had a similar association with clinicopathological markers to PIV (Table S2).

The optimal cutoff point between ‘low’ and ‘high’ values was −0.494 for ΔPIV% and −0.263 for ΔSII%. In all subtypes of breast cancer, we observed a similar distribution of low and high ΔPIV%. However, for all three molecular subtypes, more than 60% of patients belonged to the group characterized by high ΔPIV%. In contrast, patients who underwent endocrine therapy and chemotherapy had different ΔPIV%. High ΔPIV% values were associated with the chemotherapy group (Table 4, *p* = 0.024). There was also a trend toward a high ΔPIV% in higher lines and later cycles of therapy. ΔPIV% did not correlate with the presence of megakaryocytes or CTC groups. ΔSII% was associated with clinicopathological features in a similar way to ΔPIV%.

### 3.2. Overall Survival

**Figure 4:**
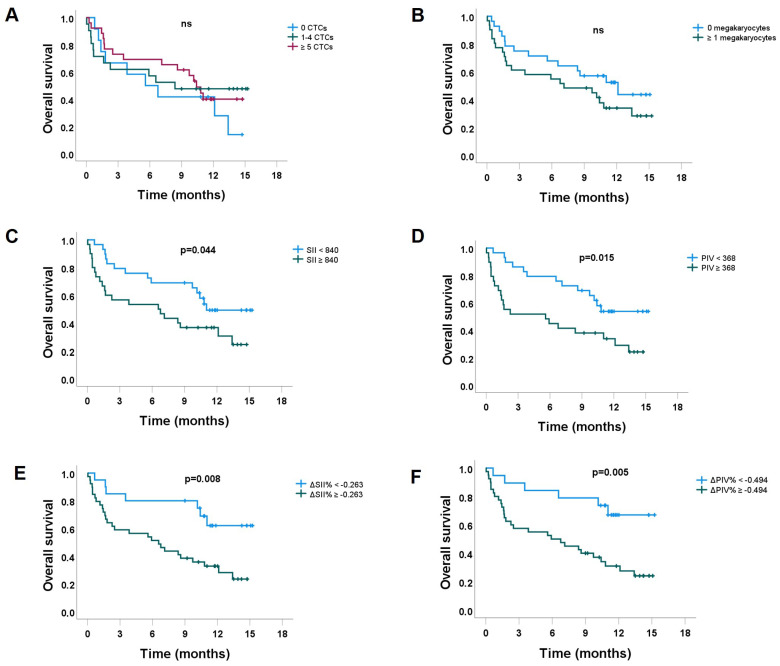
Overall survival of metastatic breast cancer patients according to the (**A**) number of circulating tumor cells (CTCs), (**B**) number of megakaryocytes 0 vs. ≥1, (**C**) low vs. high systemic immune-inflammation index (SII), (**D**) low vs. high pan-immune-inflammation value (PIV), (**E**) low vs. high relative changes in SII (ΔSII%), and (**F**) low vs. high relative changes in PIV (ΔPIV%).

We evaluated potential factors affecting OS (CTCs, megakaryocytes, PIV, SII, ΔPIV%, and ΔSII%) (Table 5). We found no difference in OS among the groups of patients characterized by 0 CTCs, 1–4 CTCs, and ≥5 CTCs (Figure 4A). The OS curves of patients with megakaryocytes present in the blood samples compared to their counterparts without megakaryocytes were clearly separated, with the former having lower OS, but the difference did not reach statistical significance (Figure 4B). Patients with low SII and low PIV had statistically significantly higher OS than their counterparts with high markers (Figure 4C,D). There was an even more pronounced difference in OS among the patients with low ΔSII% and low ΔPIV%. Both groups showed significantly higher OS than their counterparts (Figure 4E,F).

#### 3.2.1. The Impact of CTC Counts, Stratified by the Level of ΔSII% and ΔPIV%

**Figure 5:**
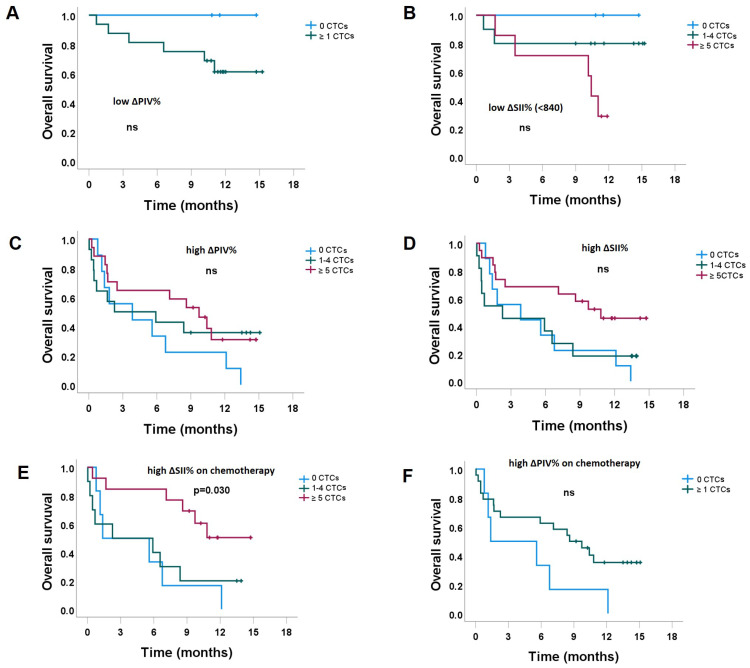
The impact of CTC count on overall survival in different immuno-inflammatory conditions: (**A**) low relative changes in PIV (ΔPIV%), (**B**) low relative changes in SII (ΔSII%), (**C**) high ΔPIV%, (**D**) high ΔSII%, (**E**) high ΔSII%, patients on treatment with chemotherapy, (**F**) high ΔPIV%, patients on treatment with chemotherapy.

We were further interested in examining whether a relative change in ΔSII% and ΔPIV% could modulate the prognostic role of CTCs on OS. Patients characterized by having low ΔPIV%, 0 CTCs vs. ≥1 CTC showed a clear separation of survival curves, while the group without CTCs tended to have better OS compared to the group characterized by having ≥1 CTCs (Figure 5A). Similarly, in the subset of patients characterized by having low ΔSII%, 0 CTCs vs. 1–4 vs. ≥5 tended to be prognostic, with a worse prognosis associated with a higher number of CTCs (Figure 5B). However, our results showed no significant differences. On the other hand, the subset of patients characterized by having high ΔPIV% and ΔSII% was not prognostically associated with the number of CTCs (Figure 5C,D). Interestingly, patients with high ΔSII% who underwent chemotherapy, especially those patients characterized by having ≥5 CTCs were significantly associated with higher OS than patients characterized by having 1–4 and 0 CTCs (Figure 5E). The same trend was observed in patients who underwent chemotherapy and had high ΔPIV% values. Specifically, patients with CTCs demonstrated better outcomes than those without CTCs (Figure 5F).

#### 3.2.2. Prognostic Factors for OS

Parameters, such as SII, PIV, ΔSII%, ΔPIV%, length of metastatic disease, and infection in the last month, were revealed as significant prognostic factors according to the results of the univariate analysis. The hazard ratios of all factors evaluated in univariate analysis of OS are presented in Table 5. ΔSII% and ΔPIV% had higher significance in univariate analysis among the immuno-inflammatory factors than SII and PIV and were used for the multivariate analysis.

**Table 5:** Univariate analysis of the prognostic factors for overall survival.

Variable	HR (95% CI)	*p* Value
Molecular subtype	1.07 (0.87–1.32)	0.530
CTCs 0 CTC vs. ≥1 CTC	0.71 (0.33–1.51)	0.373
Clusters Absent vs. present	1.05 (0.32–3.46)	0.931
Length of metastatic disease	1.38 (1.04–1.85)	0.028
Infection in the last month Yes vs. no	4.26 (1.77–10.22)	0.001
ΔSII% High vs. low	2.92 (1.27–6.719	0.012
SII High vs. low	1.98 (1.01–3.91)	0.048
ΔPIV% High vs. low	3.30 (1.36–7.99)	0.008
PIV High vs. low	2.32 (1.16–4.66)	0.018
Megakaryocytes Yes vs. no	1.60 (0.81–3.15)	0.174

CTCs: Circulating tumor cells, PIV: Pan-inflammatory value, ΔPIV%: Relative change in pan-inflammatory value, SII: Systemic immune-inflammatory index, ΔSII%: Relative change in systemic immune-inflammatory index.

In the multivariate analysis (Table 6), the duration of metastatic disease, infection, and ΔPIV% were confirmed as independent factors for the OS of the patients in our cohort. In fact, patients with a longer duration of metastatic disease had a 1.59x higher risk of death. Additionally, patients who had experienced an infection in the last month and those with high ΔPIV% values had a 4.46× and 7.88× higher risk of death, respectively.

## 4. Discussion

In the present study, we evaluated the possible correlation between CTCs, clusters, and megakaryocytes. Additionally, the prognostic significance of CTC counts, as well as megakaryocytes and immune-inflammatory markers on OS in metastatic breast cancer patients, was investigated. Our results showed a positive association between the presence of megakaryocytes and the number of CTCs, and the presence of CTC clusters with ≥5 CTCs. Our main finding was the confirmation of high ΔPIV% being a negative prognostic factor for OS for patients with metastatic breast cancer, reflecting the predominance of inflammatory cells (neutrophils, monocytes) and platelets against immune cells (lymphocytes) in these patients. Accordingly, the presence of infection in the last month, together with a longer duration of metastatic disease, were also found to be negative prognostic factors. Furthermore, we showed that a higher number of CTCs was identified as a negative prognostic factor for OS but only in the early phase of metastatic disease, which coincided with the low ΔPIV% values, i.e., with a less immunoinflammatory environment in the blood samples. If patients had more advanced metastatic disease, accompanied by high ΔPIV% or ΔSII% values, the number of CTCs no longer had prognostic significance. Another important finding, which has not yet been reported in metastatic breast cancer, is the identification of megakaryocyte presence on Giemsa CTC slides prepared after the isolation of CTCs from the blood samples of the patients. The presence of megakaryocytes was associated with a group of patients who were characterized by having a high number of CTCs (≥5), the presence of CTC clusters, and a highly immunoinflammatory blood environment (assessed by high PIV and ΔSII% values). We hypothesize that megakaryocytes in peripheral blood might have a role in facilitating or supporting the transit of CTCs within a highly immunoinflammatory environment.

In our study, we isolated CTCs using the Parsortix^®^ method. We chose this method due to its ability to capture CTCs based on their size (>6.5 μm), independent of the expression of epithelial markers on their surface, indicating that CTCs expressing nonepithelial (mesenchymal or other) markers could also be captured. Based on our previous findings, CTCs could maintain their original morphology after being separated by the Parsortix^®^ method [21] and could be subsequently stained by immunohistochemistry, which is essential for proper cytopathological evaluation. The Parsortix^®^ method has already been FDA-approved for CTC harvest in metastatic breast cancer patients and subsequent user-validated analysis. Due to its microfluid filters, this method also allows capturing not only individual CTCs but also clusters and other larger cells, such as megakaryocytes, as shown in our case. Notably, we identified at least one CTC in 79.7%, and ≥5 CTCs in 35.2% of all patients. These results are comparable to previously published data [5]. Moreover, in the HER2+ subtype, 75% of our patients had ≥5 CTCs/7.5 mL of blood. Deutsch et al., using the CellSearch^®^ method, captured at least one CTC/7.5 mL in 53.3% of patients with de-novo metastatic HER2-positive disease and in 67% of patients with HER2-negative disease [26]. They found the lowest percentage of patients with ≥1 CTC in patients on anti-HER2 therapy (28.6%). Thus, when using CTC count as an indicator for aggressive or indolent metastatic disease, information on whether the patient is currently undergoing therapy should be considered [26].

In 8.5% of all patients, we also confirmed the presence of CTC clusters. CTC clusters are defined as precursors of metastasis and are associated with higher metastatic potential than single CTCs [27]. In comparison with other studies [28,29], we found a lower percentage of patients characterized by the presence of clusters in the blood samples. One of the possible reasons for this discrepancy could be the fact that we evaluated the presence of clusters in patients who were already receiving systemic treatment and not before initiating the treatment. Wang et al. [28] detected at least one CTC cluster in 16.4% of patients with metastatic breast cancer before starting a new line of therapy. In addition to the negative prognostic factor of having ≥5 CTCs, they demonstrated that the CTC cluster and larger-size cluster added additional negative prognostic value for the OS. The main challenge is, of course, the ability to separate CTC clusters from individual CTCs since clusters can be dispersed during separation. Vetter et al. [29] demonstrated the presence of CTC clusters in 35% of patients with CTCs. The patient cohort in this study consisted of all subtypes and underwent cluster evaluation when treatment was on hold before starting the next line of treatment. Ideal platforms for the isolation of CTC clusters should allow the isolation of clusters of different sizes in an epitope-independent manner, accompanied by short processing times, with the ability to preserve the integrity of the clusters as well as recover viable cells [30].

In 52.5% of our patients, we demonstrated the presence of megakaryocytes on the evaluated Giemsa CTC slides. In addition to the observed typical morphologic characteristics observed by experienced hematocytopathologists, positive staining for CD61 and no staining for CD68 and cytokeratin antibodies additionally supported the presumption that the isolated cells were megakaryocytes, and not macrophages or CTCs. We used an anti-CD61 antibody since it is routinely used for confirmation of megakaryocytes in bone marrow samples in our pathology department. Other specific markers that could be used for confirmation of megakaryocyte presence could also be CD41 and CD42B antibodies. Notably, we also tested anti-CD41 antibody, but we chose CD61 over CD41 antibody due to its more intensive ICC staining reaction on methanol-fixed cytospins. Antibodies against CD61 (also CD41 or CD42B) rather stain the membrane or the cell surface of platelets and megakaryocytes. Positive ICC staining could also give the impression of whole-cell staining in cytology samples instead of the typical ring-shaped membrane staining pattern observed in histology samples (Figure 3C). The reason for this phenomenon is that in cytological samples, the cells are intact and not cut as in histological samples.

There seemed to be an association between the number of CTCs, CTC clusters, and megakaryocytes that contributes to a worse prognosis. Figure 4B clearly shows a worse OS when megakaryocytes were detected in the blood samples. However, due to the low number of patients in our cohort, the difference was not statistically significant. In addition, patients with a more immunosuppressive immunoinflammatory environment (high PIV and high ΔSII%) were associated with a more frequent presence of megakaryocytes in the blood samples. Most likely, we were able to identify megakaryocytes in blood samples as a result of their big size that reached the criteria for isolation by the Parsortix^®^ method we used. Namely, another method, the Cell-Search^®^ method, would detect only cells that express epithelial markers. To the best of our knowledge, this is the first report on megakaryocytes in the blood of patients with metastatic breast cancer. Megakaryocytes are normally present in bone marrow only. Rarely are they detected in blood in different kinds of diseases. Zhu et al. reported the presence of megakaryocytes in the blood in diverse nonhematological diseases (Sheehan’s syndrome, lumbar disc herniation, hypertension, Ebstein’s anomaly, dengue fever, vasculitis, myocardial infarction, cholelithiasis, pulmonary cryptococcosis, systemic lupus erythematosus), and gastric and liver cancer [18]. Similar to our findings in breast cancer, there are two other studies that detected megakaryocytes in non-small cell lung cancer [31,32] and one in prostate cancer [33]. Moreover, another study conducted in mice has provided further evidence supporting the substantial contribution of the lungs to terminal platelet production. They also found out that the lungs are responsible for approximately half of the total production of platelets. [34]. Dejima et al. reported the presence of CD61+/cytokeratin-/CD34− megakaryocytes in a cohort of patients with lung cancer. Megakaryocytes were found in pulmonary artery blood derived immediately after surgery. They probably have a role in systemic thrombopoiesis in lung cancer patients [32]. Moreover, Zhang et al. performed a global characterization of megakaryocytes. Classical megakaryocytes were found in non-small cell lung cancer and had diagnostic value. Nonclassical megakaryocyte type 2 contributes to adaptive immunity and the progression of non-small cell lung cancer [31]. In concordance with these findings, megakaryocytes in our study with metastatic breast cancer could also be interpreted as potential markers of advanced resistant disease since they positively correlated with a high immunoinflammatory blood environment, which was revealed to be a prognostic factor for shorter OS. In contrast to our findings, Xu et al. reported that patients with metastatic castration-resistant prostate cancer with ≥3 megakaryocytes are characterized with longer lifespans. However, the analysis was explorative and based on only 40 patients. More important seemed to be the score of megakaryocytes to mesenchymal CTCs, a score ≥2.0 strongly correlated to poor survival [33]. This score, however, indicates a negative prognostic value of megakaryocytes in advanced (mesenchymal CTC-positive) prostate cancer, which is in line with our findings.

Furthermore, we evaluated new prognostic indexes in metastatic breast cancer patients, such as PIV and ΔPIV%. The PIV value has been recently shown to predict OS in operable breast cancer patients [14]. In our cohort, the distribution of low and high PIV values did not depend on molecular subtypes or the number of CTCs. We found that low PIV values were significantly associated with endocrine therapy, and a trend toward skeletal metastases was also observed (Table 3). This could be explained by the fact that endocrine-dependent tumors often invade the skeleton first and are initially treated with endocrine therapy. In the course of the disease, they become resistant to endocrine therapy and need chemotherapy treatment. Hence, in patients who underwent chemotherapy, we observed high PIV values (Table 3). Patients with high SII and PIV values had worse OS (Figure 4C,D). Similar to our findings, an elevated SII was associated with a worse OS in many solid tumors [12], and among others in early breast cancer [13]. Moreover, Ligorio et al. showed that PIV outperformed other well-known peripheral blood parameters in the first-line treatment of HER2+ advanced breast cancer [35].

When evaluating the possible prognostic value of CTC count on OS in the entire cohort, we failed to demonstrate this hypothesis, either when comparing 0 vs. ≥1 CTCs, and when comparing 0 vs. 1–4 vs. ≥5 CTCs groups. This observation is rather different from the reports of others [28,36,37]. A large pooled analysis of individual patient data has already shown that the number of CTCs (≥5 vs. <5) on the CellSearch^®^ platform provides prognostic information related to OS [5]. One of the reasons why we did not confirm the prognostic role of CTCc for the entire cohort might be the fact that we included a low number of heterogeneous patients. More than half (56%) of patients were on the 3rd or later line of therapy, half had metastatic disease for more than 2 years, and in addition, in 60% of blood samples CTCs were harvested beyond the second cycle of therapy due to progression (Table 1). Despite this, we found some interesting facts, especially in relation to immune-inflammatory markers. For instance, low values of PIV, ΔSII%, and ΔPIV% were associated with skeletal metastases and endocrine treatment. In this context, a high CTC count indicated lower OS (Figure 5A,B). In contrast, CTC count did not show any prognostic correlation for the patients characterized by having high ΔSII%, and ΔPIV% (that were additionally associated with chemotherapy treatment and treatment beyond 3rd line) (Figure 5C,D). Finally, according to the multivariate analysis, the duration of metastatic disease, recent infection, and high ΔPIV% were identified as independent prognostic factors for death. In our opinion, the evaluation of ΔPIV% during the course of metastatic disease deserves more attention because of the ease of its assessment. Liquid biopsy is a promising tool that could add complementary or additional data to the radiological assessment of the disease course. However, clinical utility needs further evaluation.

A major advantage of our study is the homogeneity of the patients’ treatment and follow-up within a single oncology center. Notably, all patients were on active treatment. Furthermore, all of the slides used for CTC, cluster, and megakaryocyte evaluation were triple assessed by two independent researchers and one experienced cytopathologist and confirmed by additional ICC staining. However, there are some limitations of this study. First, we included a relatively small number of patients with heterogeneous metastatic disease, not necessarily before starting a new line of treatment, and counts of CTCs and clusters might have been influenced by effective therapy. Second, since CTC collection was performed only once, the dynamics in the counts of CTC, clusters, and megakaryocytes on OS could not be assessed. Many authors have shown the value of longitudinal monitoring of CTCs and CTC clusters during therapy. Patients who had CTC decline compared to those without CTC decline showed a more favorable prognosis [26,38,39]. Patients with CTC clusters had significantly worse survival than patients without any cluster presence [36,38]. Third, megakaryocytes could be more unequivocally confirmed by additional immunohistochemical staining.

The finding of megakaryocytes in liquid biopsy samples may have a great impact on further research. This finding raises numerous questions: How megakaryocytes enter the bloodstream, are they involved in cancer dissemination, do they retain a role in platelet production, and if so—which role they might imply to other blood cells, immune cells, CTCs, and clusters.

Studies on liquid biopsy may soon have clinical implications. Namely, immune-inflammatory indexes could be routinely calculated since blood cell measurements are routinely performed. CTC count is already an established prognostic factor for OS, however, there are no available clinical guidelines or consensus statements on the method of determination, type of staining, timeline of collection, and clinical utility. More interesting could be performing cytopathological evaluation of CTCc before and after a new line of therapy, especially when therapy will move forward to more personalized treatment, possibly in line with the other biomarkers in liquid biopsy.

## 5. Conclusions

In conclusion, the cancer disease course is largely driven by immuno-inflammatory factors in blood, which was also confirmed by our analysis. We showed that patients with low PIV and low SII at CTC collection have longer OS. Moreover, high ΔPIV% (defined as a relative change between the first metastatic spread to the evaluation point) is a negative prognostic factor for OS. This indicates that the exhaustion of an effective immune system is a driving force throughout disease progression and is even more pronounced in later stages. In addition, CTC count in metastatic breast cancer tends to be prognostic in the case of low ΔSII% and low ΔPIV% values.

Finally, we are the first to show megakaryocytes in the blood of metastatic breast cancer patients and found a positive association of megakaryocytes with CTC count, CTC clusters, and high PIV. These findings indicate a possibly important involvement of megakaryocytes in the metastatic process. However, further research is needed.

## Figures and Tables

**Table 1:** Clinical data of patients and isolated cell characteristics compared according to the number of circulating tumor cells (CTCs).

Variable	No CTCs Number (%)	1–4 CTCs Number (%)	≥5 CTCs Number (%)	All Number (%)	*p* Value
Age at CTC collection Median (IQR) years	64.9 (45.9–78.0)	55.5 (49.4–67.3)	61.3 (52.8–71.5)	60.4 (52.3–71.2)	0.255
**Subtype** Luminal A/B HER2-positive Triple-negative	8 (21.6) 1 (8.3) 3 (30)	16 (43.3) 2 (16.7) 3 (30)	13 (35.1) 9 (75) 4 (40)	37 (62.7) 12 (20.3) 10 (17.0)	0.161
**Type of therapy** Endocrine Chemotherapy	6 (31.6) 6 (15.4)	7 (36.8) 14 (35.9)	6 (31.6) 19 (48.7)	19 (32.8) 39 (67.2)	0.287
**Duration of relapse** <1 year 1–2 years 2–3 years >3 years	3 (18.8) 3 (21.4) 2 (20) 4 (21.1)	3 (18.8) 6 (42.9) 4 (40) 8 (42.1)	10 (62.4) 5 (35.7) 4 (40) 7 (36.8)	16 (27.1) 14 (23.7) 10 (17.0) 19 (32.2)	0.742
**Line of therapy** 1st and 2nd line 3rd and 4th line ≥5th line	5 (19.2) 3 (15.8) 4 (28.6)	8 (30.8) 7 (36.8) 6 (42.8)	13 (50) 9 (47.4) 4 (28.6)	26 (44.1) 19 (32.2) 14 (23.7)	0.721
**Cycle of therapy** 1st or 2nd cycle Beyond 2nd cycle	6 (25) 6 (17.2)	10 (41.7) 11 (31.4)	8 (33.3) 18 (51.4)	24 (40.7) 35 (59.3)	0.385
**Skeletal metastases** No Yes	4 (19) 8 (21.0)	6 (28.6) 15 (39.5)	11 (52.4) 15 (39.5)	21 (35.6) 38 (64.4)	0.610
**Liver metastases** No Yes	5 (10) 7 (20.6)	7 (28) 14 (41.2)	13 (51) 13 (38.2)	25 (42.4) 34 (57.6)	0.516
**Megakaryocytes** No Yes	8 (28.6) 4 (12.9)	12 (42.8) 9 (29)	8 (28.6) 18 (58.1)	28 (47.5) 31 (52.5)	0.065
**PIV at relapse** Low (<311) High (≥311)	5 (17.3) 7 (23.3)	11 (37.9) 10 (33.3)	13 (44.8) 13 (43.3)	29 (49.2) 30 (50.8)	0.834
**PIV at CTC collection** Low (<368) High (≥368)	5 (17.2) 7 (24.1)	10 (34.5) 10 (34.5)	14 (48.3) 12 (41.4)	29 (50) 29 (50)	0.784
**SII at relapse** Low (<646) High (≥464)	4 (13.8) 8 (26.7)	11 (37.9) 10 (33.3)	14 (48.3) 12 (40)	29 (49.2) 30 (50.8)	0.468
**SII at CTC collection** Low (<840) High (≥840)	4 (13.8) 8 (26.7)	11 (37.9) 10 (33.3)	14 (48.3) 12 (40)	29 (49.2) 30 (50.8)	0.468
**ΔPIV%** <−0.494 ≥−0.494	3 (15) 9 (23.1)	10 (50) 11 (28.2)	7 (35) 19 (48.7)	10 (33.9) 39 (66.1)	0.252
**ΔSII%** <−0.263 ≥−0.263	3 (15) 9 (23.1)	10 (50) 11 (28.2)	7 (35) 19 (48.7)	20 (33.9) 39 (66.1)	0.252

SII: Systemic immune-inflammation index, PIV: Pan-inflammatory value, CTCs: Circulating tumor cells.

**Table 2:** Comparison of the clinical and blood-based characteristics between the groups characterized by the presence vs. of absence of megakaryocytes in the blood samples.

Variable	No Megakaryocytes	Yes Megakaryocytes	All	*p* Value
**Subtype** Luminal A/B HER2-positive Triple-negative	18 (48.6) 5 (41.7) 5 (50)	19 (51.4) 7 (58.3) 5 (50)	37 (62.7) 12 (20.3) 10 (17.0)	0.901
**Line of therapy** 1st–2nd line 3rd–4th line ≥5th line	15 (57.7) 6 (31.6) 7 (50)	11 (42.3) 13 (68.4) 7 (50)	26 (44.1) 19 (32.2) 14 (23.7)	0.208
**Type of therapy** Endocrine Chemotherapy	11 (57.9) 17 (43.6)	8 (42.1) 22 (56.4)	19 (32.8) 39 (67.2)	0.306
**Skeletal metastases** No Yes	10 (47.6) 18 (47.4)	11 (52.4) 20 (52.6)	21 (35.6) 38 (64.4)	0.985
**CTC clusters** No Yes	28 (51.9) 0 (0)	26 (48.1) 5 (100)	54 (91.5) 5 (8.5)	0.026
**PIV at CTC collection** Low High	17 (58.6) 10 (34.5)	12 (41.4) 19 (65.5)	29 (50) 29 (50)	0.065
**SII at CTC collection** No Yes	15 (51.7) 13 (43.3)	14 (48.3) 17 (56.7)	29 (49.2) 30 (50.8)	0.519
**ΔPIV** <−0.494 ≥−0.494	11 (59.9) 17 (42.5)	8 (42.1) 23 (57.5)	19 (32.2) 40 (67.8)	0.269
**ΔSII** <−0.263 ≥−0.263	13 (65) 15 (38.5)	7 (35) 24 (61.5)	20 (33.9) 39 (66.1)	0.053

SII: Systemic immune-inflammation index, PIV: Pan-inflammatory value, CTCs: Circulating tumor cells.

**Table 3:** Comparison of clinical and blood-based characteristics between groups with high and low pan-inflammation values.

Variable	Low PIV (<368)	High PIV (≥368)	All	*p* Value
**Subtype** Luminal A/B HER2-positive Triple-negative	20 (55.6) 5 (41.7) 4 (40)	16 (44.4) 7 (58.3) 6 (60)	36 (62.1) 12 (20.7) 10 (17.2)	0.555
**Type of therapy** Endocrine Chemotherapy	13 (68.4) 16 (42.1)	6 (31.6) 22 (57.9)	19 (33.3) 38 (66.7)	0.061
**Line of therapy** 1st–2nd line 3rd–4th line ≥5th line	14 (53.8) 10 (52.6) 5 (38.5)	12 (46.2) 9 (47.4) 8 (61.5)	26 (44.8) 19 (32.8) 13 (22.4)	0.638
**Cycle of therapy** 1st–2nd cycle ≥3rd cycle	10 (41.7) 19 (55.9)	14 (58.3) 15 (44.1)	24 (41.4) 34 (58.6)	0.286
**Skeletal metastases** No Yes	6 (28.6) 23 (62.2)	15 (71.4) 14 (37.8)	21 (36.2) 37 (63.8)	0.014
**Liver metastases** No Yes	13 (52) 16 (48.5)	12 (48) 17 (51.5)	25 (43.1) 33 (56.9)	0.791
**CTC clusters** No Yes	26 (49.1) 3 (60)	27 (50.9) 2 (40)	54 (91.4) 5 (8.6)	0.640
**Megakaryocytes** No Yes	17 (63) 12 (38.7)	10 (37) 19 (61.3)	27 (46.6) 31 (53.4)	0.065
**CTC group** 0 CTC 1–4 CTC ≥5 CTC	5 (41.7) 10 (50) 14 (53.8)	7 (58.3) 10 (50) 12 (46.2)	12 (20.7) 20 (34.5) 26 (44.8)	0.784

SII: Systemic immune-inflammation index (SII), PIV: Pan-inflammatory value, CTCs: Circulating tumor cells.

**Table 4:** Comparison of clinical and blood-based characteristics between groups with high and low relative changes in PIV (ΔPIV%).

Variable	ΔPIV% < −0.494	ΔPIV% ≥ −0.494	All	*p* Value
**Subtype** Luminal A/B HER2-positive Triple-negative	14 (37.8) 3 (25) 2 (20)	23 (62.2) 9 (75) 8 (80)	37 (62.7) 12 (20.3) 10 (17.0)	0.471
**Type of therapy** Endocrine Chemotherapy	10 (52.6) 9 (23.1)	9 (47.4) 30 (76.9)	19 (32.8) 39 (67.2)	0.024
**Line of therapy** 1st–2nd line 3rd–4th line ≥5th line	11 (42.3) 7 (36.8) 1 (7.1)	15 (57.7) 12 (63.2) 13 (92.9)	26 (44.1) 19 (32.2) 14 (23.7)	0.066
**Cycle of therapy** 1st–2nd cycle ≥3rd cycle	3 (12.5) 16 (45.7)	21 (87.5) 19 (54.3)	24 (40.7) 35 (59.3)	0.010
**Skeletal metastases** No Yes	4 (19) 15 (39.5)	17 (81) 23 (60.5)	21 (35.6) 38 (64.4)	0.108
**Liver metastases** No Yes	11 (44) 8 (23.5)	14 (56) 26 (76.5)	25 (42.4) 34 (57.6)	0.096
**CTC clusters** No Yes	16 (29.6) 3 (60)	38 (70.4) 2 (40)	54 (91.5) 5 (8.5)	0.316
**Megakaryocytes** No Yes	11 (39.3) 8 (25.8)	17 (60.7) 23 (74.2)	28 (47.5) 31 (52.5)	0.296
**CTC group** 0 CTC 1–4 CTCs ≥5 CTCs	3 (25) 7 (33.3) 9 (34.6)	9 (75) 14 (66.7) 17 (65.4)	12 (20.3) 21 (35.6) 26 (44.1)	0.832

CTCs: circulating tumor cells.

**Table 6:** Multivariate analysis of the prognostic factors for overall survival.

Variable	Multivariable HR (95% CI)	*p* Value
Length of metastatic disease	1.59 (1.16–2.17)	0.004
Infection in the last month Yes vs. no	7.88 (2.88–21.6)	<0.001
ΔSII% High vs. low	1.47 (0.59–3.67)	0.406
ΔPIV% High vs. low	4.46 (1.52–13.07)	0.006

ΔPIV%: Relative change in pan-inflammatory value, ΔSII%: Relative change in systemic immune-inflammatory index.

## Data Availability

Data will be available after considering the aim of further use.

## Supplementary Materials

The following supporting information can be downloaded at: https://www.mdpi.com/article/10.3390/cancers15133397/s1. Table S1. Clinical data of patients at diagnosis; Table S2. Comparison of clinical and blood-based characteristics between groups with high and low systemic immune-inflammation index; Figure S1. Correlation between the number of megakaryocytes and the number of circulating tumor cells; Figure S2. ROC curve for pan-inflammation value (PIV) (A) and systemic immune-inflammation index (SII).
