# Supplementary material for: Association of Circulating Tumor Cells, Megakaryocytes and a High Immune-Inflammatory Environment in Metastatic Breast Cancer
Source: Cancers (Basel). 2023 Jun 28;15(13):3397. doi: 10.3390/cancers15133397 (PMC10340574; doi:10.3390/cancers15133397)
Supplement: Supplementary file 1 [file cancers-15-03397-s001.zip › cancers-2296517-supplementary.pdf]

Table S1. Clinical data of patients at diagnosis

Variable		Number (%)
All patients		59 (100)
Age at cancer diagnosis (years)		
Median (SEM), range	50.3 (1.61), 24.8-81.8	
Histology	Invasive ductal carcinoma	48 (81.3)
	Invasive lobular carcinoma	6 (10.2)
	Another invasive carcinoma	4 (6.8)
	Ductal carcinoma in situ	1 (1.7)
Grade	Grade 1	3 (5.1)
	Grade 2	17 (28.8)
	Grade 3	36 (61.0)
	Unknown grade	3 (5.1)
Subtype	Luminal-like	37 (62.7)
	HER2-positive	12 (20.3)
	Triple negative	10 (17.0)
Tumor stage	T1 (≤ 20 mm)	11 (18.7)
	T2 (> 20 mm in ≤ 50 mm)	32 (54.2)
	T3 (> 50 mm)	16 (27.1)
Nodal stage	pN0	11 (18.7)
	pN1 (1-3 positive nodes)	16 (27.1)
	pN2 (4-6 positive nodes)	6 (10.2)
	pN3 (>6 positive nodes)	15 (25.4)
	No pathological stage	11 (18.7)
M stage	M0	46 (78.0)
	M1 (primary metastatic)	13 (22.0)
Surgery	Mastectomy and axillary dissection	24 (40.7)
	Mastectomy and SNB	4 (6.8)
	Breast conserving surgery and axillary dissection	10 (16.9)
	Breast conserving surgery and SNB	7 (11.9)
	Hygienic breast ablation	1 (1.7)
	No surgery performed	13 (22.0)
Adjuvant treatment	(Neo)adjuvant chemotherapy	34 (57.6)
	Adjuvant radiation therapy	39 (66.1)
	Adjuvant endocrine therapy	32 (64.2)

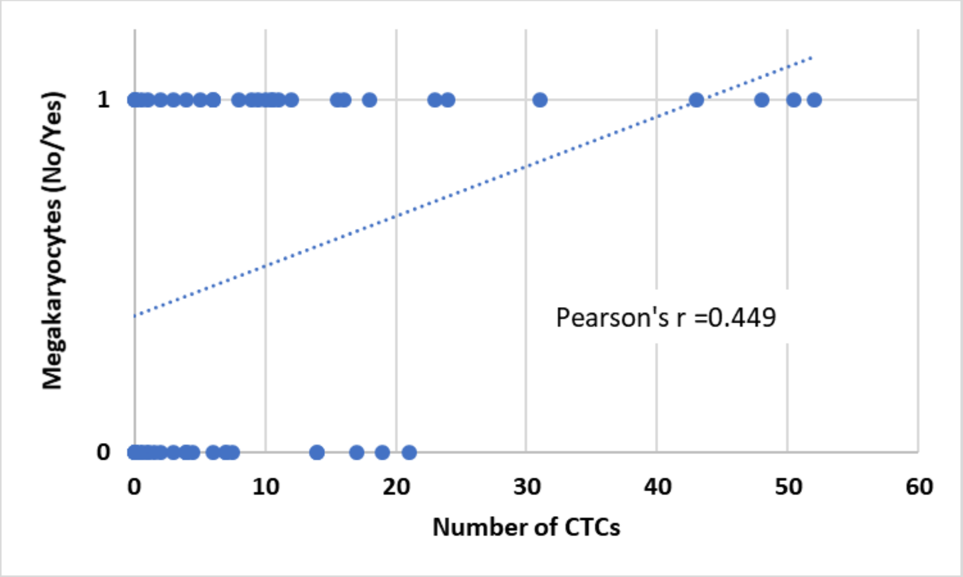

Figure S1. Correlation between the number of megakaryocytes and the number of circulating tumor cells (CTCs)

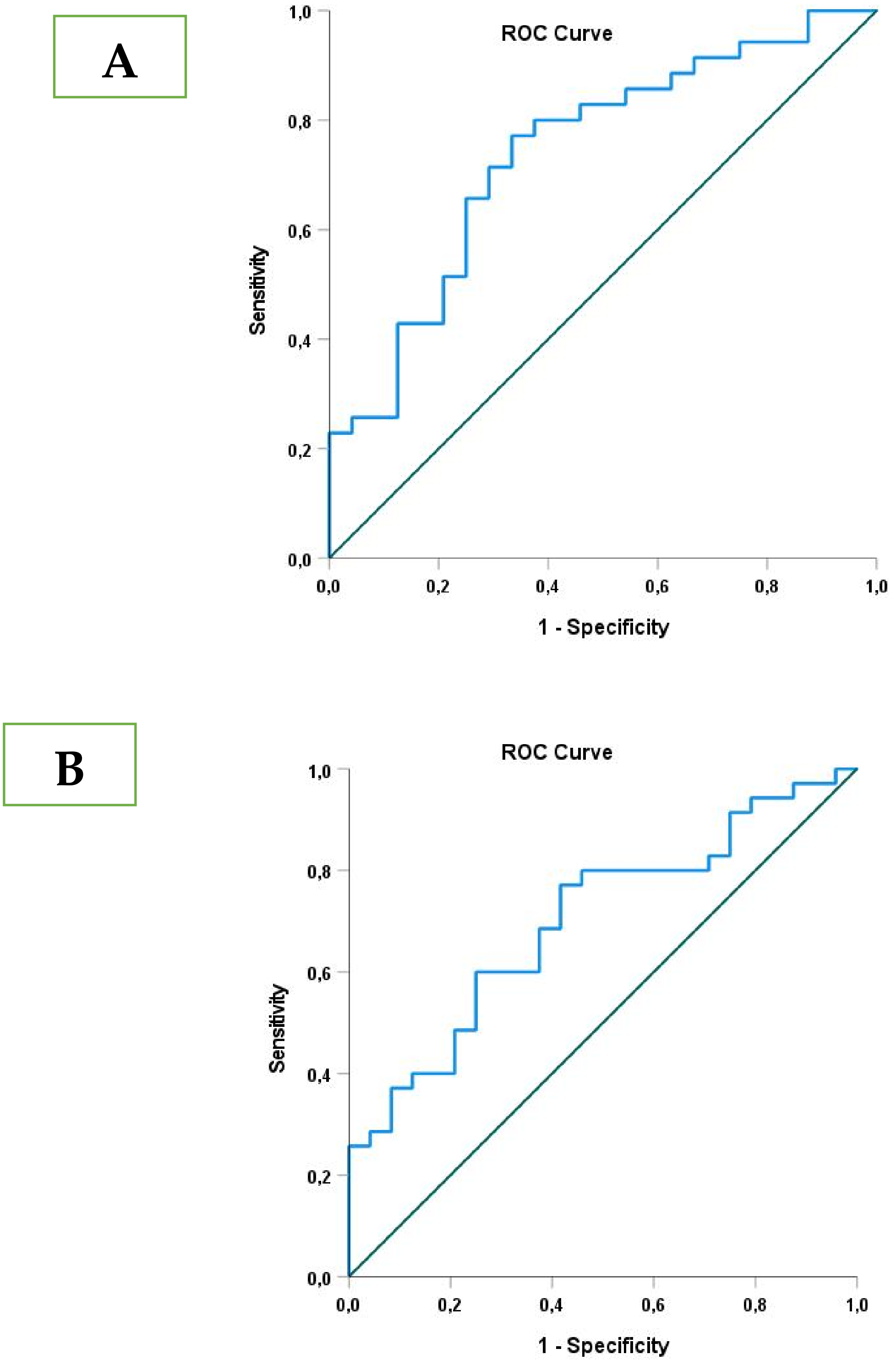

Figure S2. ROC curves) **(A)** for pan-inflammation value (PIV) and **(A)** systemic immune-inflammation index (SII).

The optimal cutoff value between the 'low' and 'high' PIV values was set at 368 and between 'low' and 'high' SII values at 840 × 10^9

Table S2. Comparison of clinical and blood-based characteristics between groups with high and low systemic immune-inflammation index.

Variable	Low SII (<840)	High SII (≥840)	All	P-value
**Subtype**				
Luminal A/B	19 (51.4)	18 (48.6)	37 (62.7)	0.815
HER2 positive	6 (50.0)	6 (50.0)	12 (20.3)	
Triple negative	4 (40.0)	6 (50.0)	10 (16.9)	
**Type of therapy**				
Endocrine	12 (63,2)	7 (36.8)	19 (32.8)	0.113
Chemotherapy	16 (41.0)	23 (59.0)	39 (67.2)	
**Line of therapy**				
1st-2nd line	15 (57.7)	11 (42.3)	26 (49.1)	0.408
3rd-4th line	9 (47.4)	10 (52.6)	19 (32.2)	
≥5th line	5 (35.7)	9 (64.3)	14 (23.7)	
**Cycle of therapy**				
1th-2nd cycle	9 (37.5)	15 (62.5)	24 (40.7)	0.138
≥3rd cycle	20 (57.1)	15 (42.9)	35 (59.3)	
**Skeletal metastases**				
No	6 (28.6)	15 (71.4)	21 (36.2)	**0.019**
Yes	23 (79.3)	14 (37.8)	37 (63.8)	
**Liver metastases**				
No	15 (60.0)	10 (40.0)	25 (42.4)	0.153
Yes	14 (41.2)	20 (58.8)	34 (57.6)	
**CTC clusters**				
No	26 (48.1)	28 (51.9)	54 (91.5)	0.612
Yes	3 (60.0)	2 (20)	5 (8.5)	
**Megakaryocytes**				
No	15 (63.6)	13 (46.4)	28 (47.5)	0.519
Yes	14 (45.2)	17 (54.8)	31 (52.5)	
**CTC group**				
0 CTC	4 (33.3)	8 (66.7)	12 (20.3)	0.468
1-4 CTC	11 (52.4)	10 (47.6)	21 (35.6)	
≥5 CTC	14 (53.8)	12 (46.2)	26 (44.1)	

SII: Systemic immune-inflammation index (SII), CTCs: Circulating tumor cells.
